# The charter on the rights of hospitalized children with disability

**DOI:** 10.1186/1824-7288-41-S2-A52

**Published:** 2015-09-30

**Authors:** Nicola Panocchia

**Affiliations:** 1 Servizio Emodialisi, Policlinico A. Gemelli, Roma, 00168, Italy; 2 Cooperativa Sociale Spes contra Spem, Cooperativa Sociale, 00139, Italy

## 

As the United Nation Convention on the Rights of Persons with Disabilities states, People with disabilities have the same general health care needs as other patients, but they are more like to find health care providers’ skill and facilities inadequate, to be denied health care, and to be treated badly in the health care systems [1]. In the United Kingdom several inquires report deaths of people with a learning disability, as consequences of healthcare inequalities [see *https://www.mencap.org.uk/get-involved/campaigns/successes/our-fight-equal-healthcare*].

Spes contra spem, a non profit organization, processed the charter of rights of people with disabilities in hospital [2]. The Charter shapes the rights set down in the European Charter of patients’ rights for people with disabilities.

The purpose was to have a tool to overcome the “sanitary barriers": architectural, organizational and cultural. It based on the principle that people with disability do not have special rights but equal rights as the others people have. Only The ways of benefit of these rights are different, and it is a duty of society to put everyone in a position to be able to benefit, by removing the barriers that stand in the way. This is the application of the principles of equality and non-discrimination of United Nation Convention [3].

Recently, we evaluated the need for a specific charter of the rights of children with disabilities in hospital. Children with disabilities have some distinctive features compared to their peers without disability that should be highlighted.

As well as should be considered the cultural barriers about the disability, present in the care of children too. It is important that health professionals understand that disability in itself is not sufficient to deny a treatment or a cure. Frequently, inadequate medical treatment of children with disability is related to the belief that they have a poor quality of life [4].

So we decided to promote the charter on the rights of hospitalized children with disability. We decided to use an existing charter, la Carta dei diritti del bambino in Ospedale drawn up by “Fondazione per il Bambino in ospedale (ABIO)” and Italian Society of Pediatrics. The ten articles, unchanged in their formulation, should be declined taking due account the needs of children with disabilities and their parents.

We propose a road map for a development of the Chapter that should be the result of the broadest possible sharing between pediatricians, nurses, the associations of disabled people and their families.
